# Suppression of the inflammatory response by disease-inducible interleukin-10 gene therapy in a three-dimensional micromass model of the human synovial membrane

**DOI:** 10.1186/s13075-016-1083-1

**Published:** 2016-08-12

**Authors:** Mathijs G. A. Broeren, Marieke de Vries, Miranda B. Bennink, Onno J. Arntz, Peter L. E M. van Lent, Peter M. van der Kraan, Wim B. van den Berg, Frank H. J. van den Hoogen, Marije I. Koenders, Fons A. J. van de Loo

**Affiliations:** Experimental Rheumatology, Department of Rheumatology, Radboud Institute for Molecular Life Sciences, Radboud University Medical Center, PO Box 9101, 6500 HB Nijmegen, The Netherlands

**Keywords:** Osteoarthritis, Gene therapy, Cytokines, Micromasses, Synovium, Inflammation

## Abstract

**Background:**

Gene therapy has the potential to provide long-term production of therapeutic proteins in the joints of osteoarthritis (OA) patients. The objective of this study was to analyse the therapeutic potential of disease-inducible expression of anti-inflammatory interleukin-10 (IL-10) in the three-dimensional micromass model of the human synovial membrane.

**Methods:**

Synovial tissue samples from OA patients were digested and the cells were mixed with Matrigel to obtain 3D micromasses. The *CXCL10* promoter combined with the firefly luciferase reporter in a lentiviral vector was used to determine the response of the *CXCL10* promoter to tumour necrosis factor alpha (TNF-α), interleukin-1β (IL-1β) and lipopolysaccharide (LPS). The effects of recombinant IL-10 on gene expression were determined by quantitative PCR. The production of IL-10 from the CXCL10p-IL10 vector and the effects on pro-inflammatory cytokine production were assessed by multiplex ELISA.

**Results:**

Micromasses made from whole synovial membrane cell suspensions form a distinct surface composition containing macrophage and fibroblast-like synoviocytes thus mimicking the synovial lining. This lining can be transduced by lentiviruses and allow CXCL-10 promoter-regulated transgene expression. Adequate amounts of IL-10 transgene were produced after stimulation with pro-inflammatory factors able to reduce the production of synovial IL-1β and IL-6.

**Conclusions:**

Synovial micromasses are a suitable model to test disease-regulated gene therapy approaches and the CXCL10p-IL10 vector might be a good candidate to decrease the inflammatory response implicated in the pathogenesis of OA.

**Electronic supplementary material:**

The online version of this article (doi:10.1186/s13075-016-1083-1) contains supplementary material, which is available to authorized users.

## Background

Osteoarthritis (OA) is the most common joint disease and no adequate disease-modifying treatments are available yet [1]. Although the exact etiology of OA is still unclear and may be dependent on multiple risk factors, there is increasing evidence that in many OA patients inflammation is involved in the pathogenesis [2]. Interleukin-1β (IL-1β) and tumour necrosis factor alpha (TNF-α) are key pro-inflammatory cytokines, primarily produced by macrophages that infiltrate the synovium during inflammatory OA [3]. These cytokines trigger the release of other cytokines and matrix-degrading enzymes from resident articular cells, including the fibroblast-like synoviocytes (FLS) that damage the articular cartilage. This results in the release of damage-associated molecular patterns (DAMPs) with pro-inflammatory properties, potentially creating a positive vicious circle of inflammation and damage [4].

Several strategies have been explored to treat inflammation in the OA joint, including biological therapies that were developed for the treatment of rheumatoid arthritis (RA). However, the therapeutic effects are disappointing [5]. A possible explanation might be the relatively strong contribution of systemic factors in RA. In contrast, OA is considered primarily to be a local process and systemically injected therapeutics may not reach the target joints in sufficient amounts. Intra-articular injections are feasible but proteins injected in the joint are rapidly cleared and consequently the invasive injections have to be repeated increasing the discomfort for patients [6]. These considerations ask for the development of a different strategy to obtain long-term drug effects in the OA joint. Local gene therapy can be a suitable approach and might even be used to express proteins with a short half-life like interleukin-10 (IL-10), which is a potent anti-inflammatory cytokine [7].

Previous studies with IL-10 gene therapy in experimental models of rheumatoid arthritis have explored the use of inducible promoters to drive the expression of IL-10 [8–10]. These promoters contain binding sites for transcription factors that are activated during active disease, which might reduce side effects of continuous IL-10 exposure as observed in patients suffering from chronic active Crohn’s disease. These patients received daily injections of high-dose IL-10 and showed a drop in haemoglobin levels [11]. We recently adapted the inducible IL-10 gene therapy to human synovial cells [12]. For this purpose, the promoter from the *CXCL10* gene was selected based on microarray analysis of RA synovium. The inflammatory C-X-C motif chemokine 10 (CXCL10) protein concentration has also been found to be significantly upregulated in the synovial fluid and in the serum of OA patients compared to healthy controls [13, 14]. Because CXCL10 can be expressed from multiple cell types, no selective expression is expected from the vector [15]. CXCL10 expression is associated with OA-related disease processes, including inflammation and osteoclastogenesis [15], which indicates that *CXCL10* promoter-driven expression of IL-10 might be a viable option for the treatment of OA. In addition, the OA synovium could be more sensitive to IL-10 therapy, because of relatively high expression of the IL-10 receptor alpha chain as compared to RA [16].

The CXCL10p-IL10 gene therapy approach showed promising results in synovial cell suspensions. Based on the known IL-10 effects we postulate that local IL-10 gene therapy would be efficacious at the early stage of OA when synovitis is developing and before irreversible fibrotic changes occur. In this study, we determined the inflammatory response and anti-inflammatory potential of the CXCL10p-IL10 lentiviral vector in the three-dimensional (3D) micromass synovial membrane model. In a 3D culture model, the cell-matrix and cell-cell interactions are more biologically relevant, providing a more predictive system for the in vivo situation, compared to classic two-dimensional (2D) culture [17]. Synovial micromasses were generated from primary synovial cells isolated from OA patients by digestion, containing both FLS and macrophage-like synoviocytes (MLS). The micromasses were transduced after establishment of a synovial lining layer and the *CXCL10* promoter was responsive to lipopolysaccharide (LPS), TNF-α and IL-1β. The activated promoter could provide therapeutic quantities of IL-10, which reduced the release of IL-1β and IL-6. These results show that the CXCL10p-IL10 vector can provide self-regulated inhibition of the inflammatory response in a synovial membrane model.

## Methods

### Patient material

**Fig. 1:**
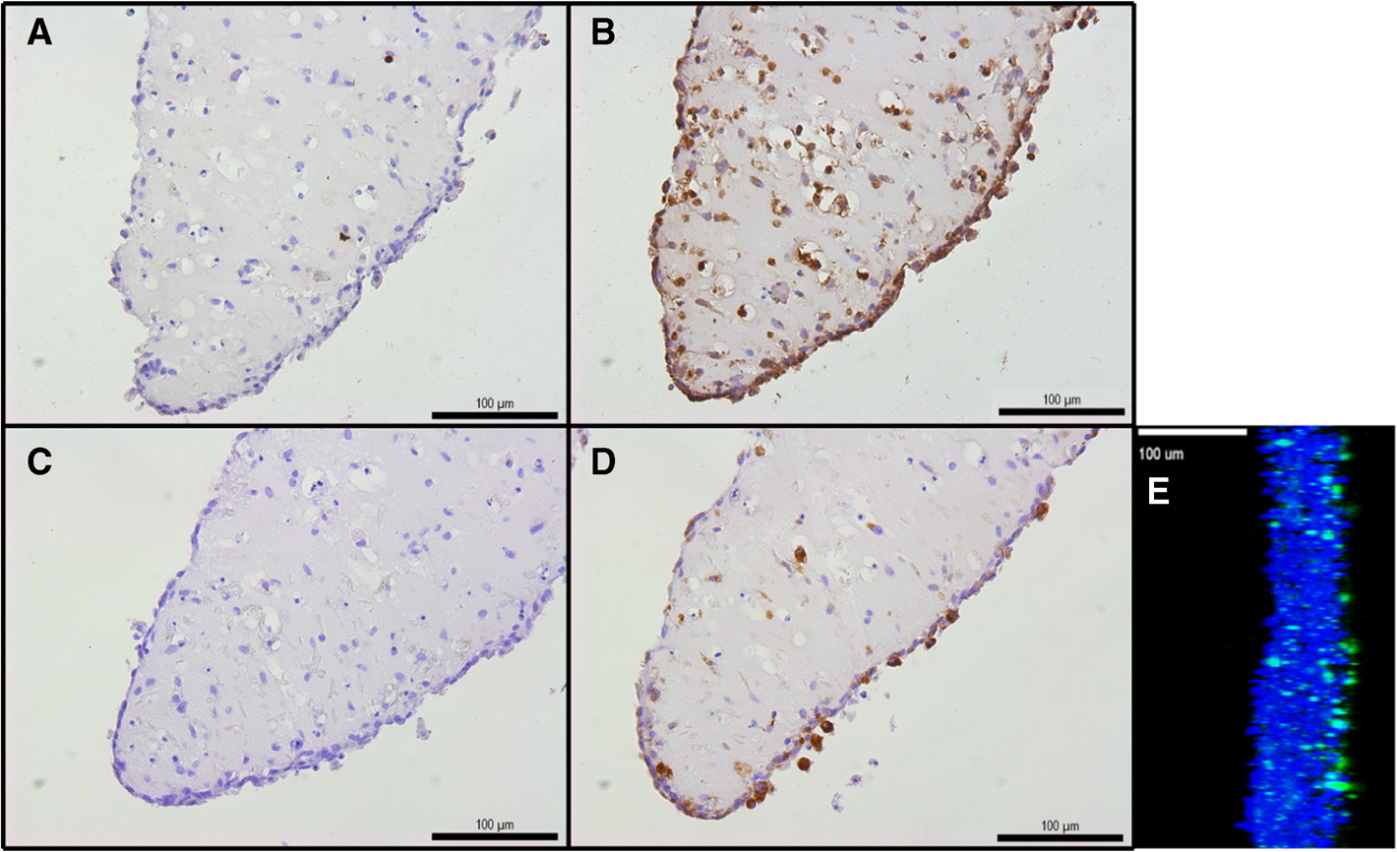
Immunohistochemical detection of fibroblasts and macrophages in synovial micromasses. Synovial micromasses were generated from digested synovial tissue cell suspension and cultured for 7 days. **a** IgG control antibody for 11-Fibrau staining. **b** 11-fibrau (*brown*) staining for synovial fibroblasts. **c** IgG control antibody for CD68 staining. **d** CD68 staining (*brown*) for macrophages. **e** Confocal fluorescent image of the micromass after transduction with lentiviral PGK-GFP. Side view with DAPI (*blue*) and GFP (*green*) staining. The micromasses used in Fig. 1a-d were derived from RA material and similar results were obtained staining OA micromass sections

Synovial osteoarthritis (OA) tissue samples were obtained during joint replacement surgery from the Department of Orthopedics (Radboud University Medical Center, Nijmegen, The Netherlands). Patients gave their informed consent and protocols were approved by the medical ethics committee. In total, synovium of 12 OA patients was included in this study. The micromasses shown in Fig. 1a-d were derived from a patient diagnosed with RA. Before processing, representative samples were embedded in Tissue-Tek O.C.T. (Sakura, Alphen a/d Rijn, The Netherlands). Cryosections 7 μm thick were cut using the Cryostar NX70 (Thermo Fisher Scientific, Waltham, MA, USA) and stained for hematoxylin and eosin (H&E) to confirm that the tissue samples contained a synovial lining. Additional file 1: Figure S1 contains H&E images of 12 patients.

The synovial samples were digested using 50 μg/ml Liberase TM (Roche, Basel, Switzerland) for 1 h at 37 °C in Roswell Park Memorial Institute (RPMI) culture medium without supplementations. The digestion was stopped by adding 10 % fetal calf serum (FCS). Subsequently, the synovial cells were passed through a 70-μm cell strainer (Corning, NY, USA) and centrifuged. All cell centrifugations were performed for 5 min at 1500 rpm/423 g in a Heraeus Megafuge 16R (Thermo Fisher Scientific). Red blood cells were lysed for 2 min at RT using 4 ml RBC lysis buffer (155 nM NH_4_Cl, 12 mM KHCO_3_, 0.1 mM EDTA, pH 7.3). The lysis reaction was quenched by adding 6 ml RPMI culture medium, supplemented with 10 % FCS and 1 mM pyruvate and 1 % P/S.

### Micromass production and culture

For micromass construction, the synovial cell suspensions were centrifuged and cell pellets were dissolved in ice-cold Matrigel (Corning) at an average density of 2 × 10E7 cells/ml. Using cooled pipette tips, 25 μl droplets were placed in 24-well culture plates (Greiner Bio-one, Alphen a/d Rijn, The Netherlands) or conical 12 ml tubes (Greiner Bio-one), which were coated with poly-(2-hydroxyethyl methacrylate) (poly-HEMA) (Sigma-Aldrich, Zwijndrecht, The Netherlands). After 30 minutes gelation at 37 °C, 500 μl RPMI culture medium, supplemented with 10 % heat-inactivated FCS, 1 mM pyruvate and 1 % P/S was added. Medium was replaced twice weekly. All cell cultures were kept in humidified atmosphere at 37 °C and 5 % CO_2_. The micromasses were stimulated with *E. coli* lipopolysaccharide (LPS) (Invivogen, San Diego, CA, USA), recombinant human TNF-α (Abcam, Cambridge, UK), recombinant human IL-1β (R&D systems, Oxford, UK) and recombinant human IL-10 (Life Technologies Europe, Bleiswijk, The Netherlands) at concentrations and timing as indicated in the text.

### Micromass immunohistochemistry

For immunohistochemical analysis, micromasses were fixated for 2 h in 2 % paraformaldehyde in phosphate-buffered saline (PBS)/1 mM CaCl_2,_ dehydrated and embedded in paraffin. Sections 7-μm thick were deparaffinized, rehydrated and incubated with antibodies 11-fibrau (1:100 for 60 minutes) (clone D7-fib, Imgen, distributed by ITK Diagnostics, Uithoorn, The Netherlands), mouse anti-human cluster of differentiation 68 (CD68) (1:100 for 60 minutes) (M0814, DAKO, Heverlee, Belgium) or control mouse IgG2ak (X0943, DAKO) and IgG1k (X0931, DAKO) respectively. Endogenous peroxidase activity was blocked with 3 % H_2_O_2_ (Merck Millipore, Amsterdam, The Netherlands) in methanol. Subsequently, the sections were incubated with the secondary horseradish peroxidase (HRP)-conjugated rabbit-anti-mouse IgA/G/M (1:200 for 60 minutes) (P0260, DAKO). Peroxidase was developed with diaminobenzidine and counterstained with hematoxylin for 60 seconds.

### Plasmid cloning and lentivirus production

For the production of lentiviral vectors, we made use of the third-generation self-inactivating lentiviral (SIN) vector system. The vector for pRRL-cPPT-CXCL10p-IL10-PRE-SIN has been described previously [12]. To obtain the pRRL-cPPT-PGK-IL10-PRE-SIN vector, the phosphoglycerate kinase (PGK) luciferase vector from our previous studies [18] was predigested and the CXCL10p-IL10 vector was digested with SalI and NheI (New England Biolabs, Ipswich, MA, USA) and the IL-10 gene was ligated in the predigested PGK promoter vector. For generation of the CXCL10p-fluc-RPL22p-rluc dual-luciferase vector, a new multiple cloning site (MCS) was inserted in the SIN vector between the SalI and NheI sites, containing restriction sequences for XhoI-AgeI-SpeI-PmeI-AfeI-AscI-SalI-HpaI-NheI. The XhoI overhang from the MCS was compatible with the SalI overhang from the SIN vector. The MSC was used to clone the *RPL22* promoter (cloned from human gDNA (Promega), the renilla luciferase gene from the pMCS-green Renilla luciferase vector (Thermo Fisher Scientific, Waltham, MA, USA) and the *CXCL10* promoter in one construct. The promoter from the *RPL22* gene was selected based on low variability in multiple microarrays [19]. All primer sequences are listed in Table 1. The lentivirus production, purification and quantification were performed as described previously [12]. Transduction of micromasses was performed with 150 ng virus/micromass in complete RPMI medium supplemented with 8 μg/ml polybrene (Sigma-Aldrich).

**Table 1:** List of oligonucleotide primer sequence

Oligo description	Sequence (5′ → 3′)
MCS_oligoA	TCGAGACCGGTACTAGTGTTTAAACAGCGCTGGCGCGCCGTCGACGTTAACG
MCS_oligoB	CTAGCGTTAACGTCGACGGCGCGCCAGCGCTGTTTAAACACTAGTACCGGTC
RPL22_prom_FW	TTTTACTAGTGGCGGCCTGGCTACAGCAAA
RPL22_prom_RV	TTTTGGATCCGGCGGCAGCGGAGTTAGAAAG
GAPDH_qPCR_FW	ATCTTCTTTTGCGTCGCCAG
GAPDH_qPCR_RV	TTCCCCATGGTGTCTGAGC
SOCS3_qPCR_FW	TCGGACCAGCGCCACTT
SOCS3_qPCR_RV	CACTGGATGCGCAGGTTCT
TNFa_qPCR_FW	TCTTCTCGAACCCCGAGTGA
TNFa_qPCR_RV	CCTCTGATGGCACCACCAG
IL-1b_qPCR_FW	TGGGTAATTTTTGGGATCTACACTCT
IL-1b_qPCR_RV	AATCTGTACCTGTCCTGCGTGTT

### Confocal microscopy

For confocal microscopy, day 9 micromasses produced from an OA patient were transduced with lentiviral PGK-GFP and fixed in 1 % paraformaldehyde, 48 h after transduction. Micromasses were stained with DAPI (Molecular Probes, Eugene, OR, USA) at a concentration of 0.2 ug/ml in PBS for 10 minutes, followed by three washing steps in PBS. Micromasses were mounted in Fluoromount-G (Southern Biotech, Birmingham, AL, USA). Confocal pictures were taken at ×200 magnification using the Olympus Fluoview FV1000 laser scanning microscope (Olympus, Zoeterwoude, The Netherlands). 4′,6-diamidino-2-phenylindole (DAPI) and green fluorescent protein (GFP) were imaged using lasers at excitation wavelengths of 405 nm and 488 nm respectively. Image processing was performed using the Fluoview Viewer Software V4.1 (Olympus).

### Flow cytometry

For flow cytometry analysis, micromasses were melted on ice for 2 h, 48 h after transduction with lentiviral PGK-GFP. The cell suspension was incubated with antibodies 11-Fibrau and subsequently with donkey-anti-mouse conjugated to Alexa Fluor 568 (1:100 for 30 minutes) (A-10037, Thermo Fisher Scientific), or cell suspensions were incubated with mouse-anti-human CD68, conjugated to PE (1:20 for 60 minutes) (12-0689, eBioscience, San Diego, CA, USA). The flow cytometry was performed on the FACS Cyan (Beckman Coulter, Woerden, The Netherlands) using the 488 nm laser at the FL1, FL3 and FL7 channels for GFP, 11-Fibrau and CD68 respectively.

### Luciferase measurements

The luciferase measurements were performed in 96-well white clear-bottom plates (Greiner Bio-one) using the Dual-Glo luciferase assay system (Promega). Micromasses were transferred to a well and lysed in Dual-Glo reagent for 10 minutes. Firefly luciferase light production was measured on a Clariostar (BMG, Offenburg, Germany). Subsequently, the lysates were incubated for 10 minutes with Stop & Glo before measuring the Renilla luciferase light production. The values were corrected for the background signal and depicted as relative light units (RLU).

### RNA isolation and quantitative polymerase chain reaction (qPCR)

RNA isolation and qPCR were performed as described previously [20]. The primer sequences are listed in Table 1.

### Multiplex enzyme-linked immunosorbent assay (ELISA) assay

Cytokine concentrations were determined by luminex multianalyte technology on the Bio-Plex 200 (Bio-Rad, Hercules, CA, USA) in combination with Bio-Plex pro human cytokine kits (Bio-Rad) according to the manufacturer’s protocol. For IL-10 and interleukin-6 (IL-6) measurements, the micromass culture supernatants were first diluted 25 times. Samples below the detection limit were set at the lowest measureable quantity to perform statistical analysis.

### Statistical analysis

Statistical analysis was performed using the Student’s *t* test, one-way analysis of variance (ANOVA) and two-way ANOVA. Results are depicted as mean +/- SD and *P* values < 0.05 were regarded as significant. For statistical comparisons between conditions including multiple patients, patients were first individually normalized for the control conditions.

## Results

### The synovial micromass membrane contains FLS and MLS

In previous studies that used the synovial micromass model, primary FLS were used that had been cultured for multiple passages. However, sustained culture of FLS can result in phenotype alterations [20] and in FLS micromasses the contribution of MLS is omitted. Therefore, we first tested the ability of synovial cell suspensions derived from digested synovium to form a lining layer in micromass culture. After 7 days, cells had migrated to the micromass-medium interface and resembled a synovial membrane (Fig. 1). The sections were stained using the 11-fibrau antibody, which revealed that most cells in the lining are synovial fibroblast-like cells (Fig. 1b). In addition, we performed a staining for the macrophage marker CD68. Macrophages were also present in the micromasses and appeared in the lining at day 7 (Fig. 1d). This shows that the synovial micromass model can be used to study a membrane that resembles the architecture and composition of the synovial lining. When the micromasses were transduced with a lentiviral PGK-GFP vector, GFP expression was mainly observed in the lining layer (Fig. 1e) and to a lesser extent in the sublining. The transduction efficiency and virus tropism were assessed using flow cytometry. Around 6 % of the cells expressed GFP after transduction and both FLS (6.7 % of the 78.7 % 11-fibrau-positive cells) and MLS (5.3 % of the 38.0 % CD68-positive cells) were transduced (Additional file 2: Figure S2).

### The *CXCL10* promoter responsiveness in synovial micromasses

**Fig. 2:**
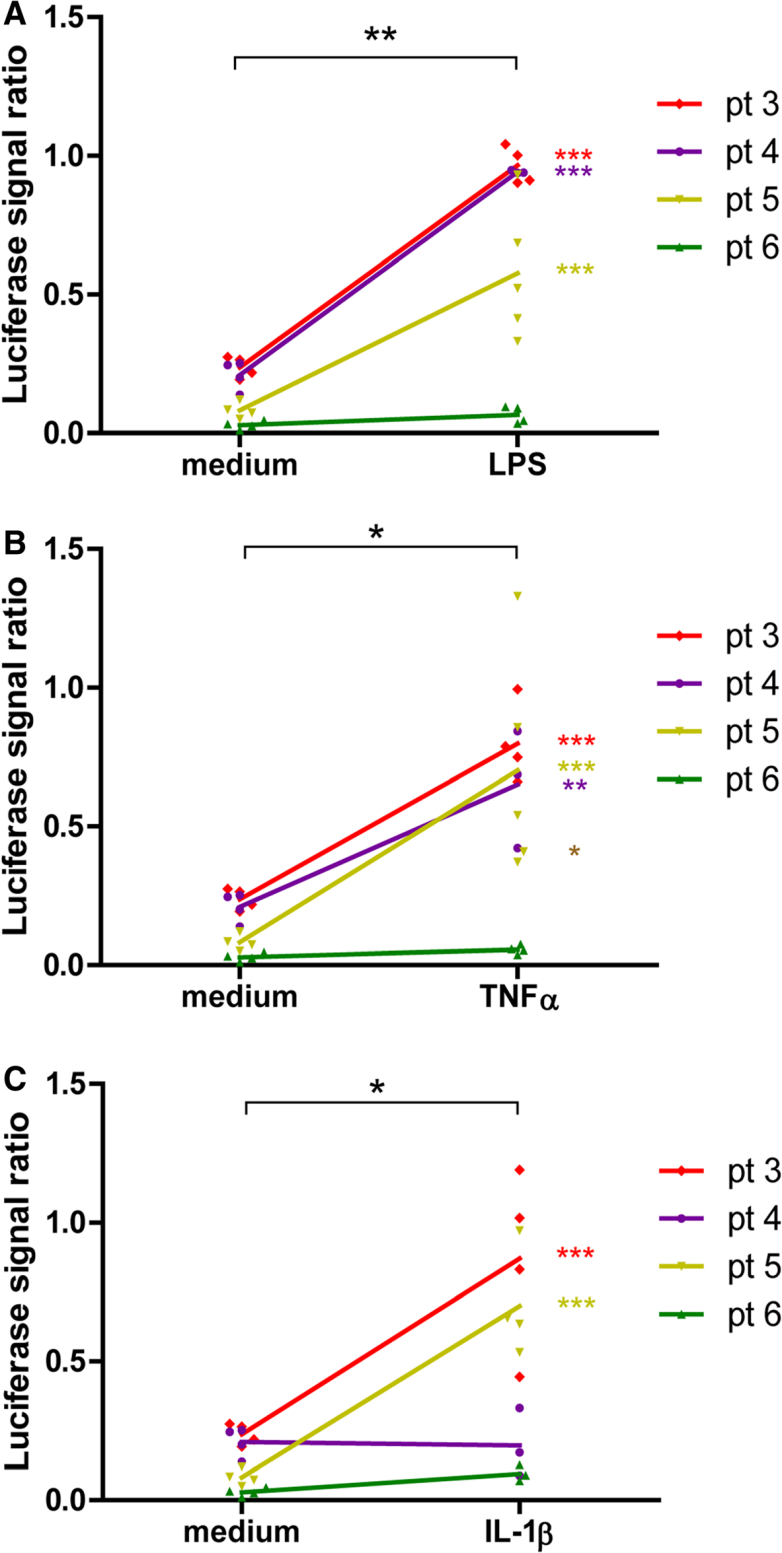
Activation of the *CXCL10* promoter in synovial cell micromasses by LPS, TNF-α and IL-1β. Synovial micromasses of four OA patients were transduced with the CXCL10p-fluc-RPL22p-rluc dual-luciferase construct and stimulated for 6 h with (**a**) 100 ng/ml LPS, (**b**) 10 ng/ml TNF-α or (**c**) 10 ng/ml IL-1β and compared to the unstimulated medium condition. The signal ratio was calculated as fireflyRLU/renillaRLU +/- SD. Micromasses are depicted as individual points and different colours represent different patients. Statistical analysis between medium and stimulated condition was performed by Student’s *t* test and comparisons within individual patient samples were calculated by two-way ANOVA. *P < 0.05, **P < 0.01. *IL-1β* interleukin-1 beta, *LPS* lipopolysaccharide, *TNF-α* tumour necrosis factor alpha

The micromasses were transduced with a lentiviral vector to study the response of the *CXCL10* promoter, the promoter of our choice to obtain autoregulated transgene expression. The vector contained a CXCL10p-firefly luciferase reporter and an 60S ribosomal protein L22 (RPL22p)-renilla luciferase gene to correct for micromass cellularity and transduction efficiency. Micromasses were transduced after forming a lining to resemble the in vivo synovial membrane transduction and stimulated with LPS, TNF-α or IL-1β for 6 hours. Previous studies have found an upregulation of multiple plasma proteins in the OA synovial fluid, which similar to DAMPs can activate macrophages in a TLR-4-dependent manner [13]. We therefore included the TLR-4-specific stimulus LPS in our studies. Although not significant for every individual patient, stimulation with LPS, TNF-α or IL-1β resulted in a significant upregulation of *CXCL10* promoter activity (Fig. 2a-c). These results show that the cells in the micromass lining can be transduced by lentiviruses and disease-related triggers can activate the *CXCL10* promoter. Interestingly, one patient (OA4) showed no significant response to any of the stimuli.

### The effects of IL-10 protein on synovial micromasses

**Fig. 3:**
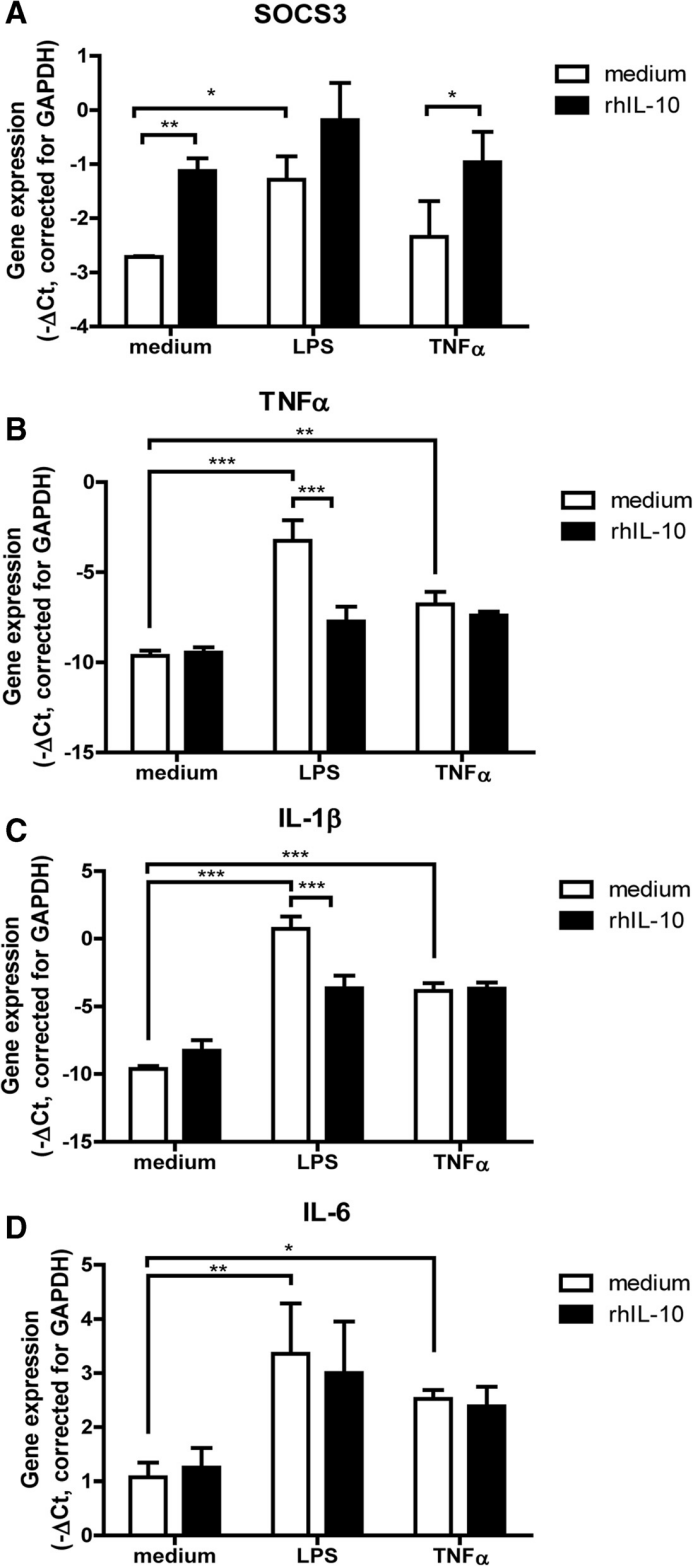
Gene expression in synovial micromasses after stimulation with LPS and TNF-α in the absence or presence of IL-10. Micromasses from synovial cell suspensions (three per group) were cultured until lining formation was evident. Subsequently, the micromasses were stimulated for 2 h or 4 h with medium containing 100 ng/ml LPS, 10 ng/ml TNF-α, 10 ng/ml IL-1β in the absence and presence of 10 ng/ml IL-10. Gene expression levels of SOCS3 at 2 h (**a**), TNF-α at 4 h (**b**), IL-1β at 4 h (**c**) and IL-6 at 4 h (**d**) were measured. Expression levels are depicted as threshold cycle (Ct) +/- SD, corrected for GAPDH expression. Statistical comparison within stimulation groups was performed by two-way ANOVA and between groups by one-way ANOVA. *P < 0.05, **P < 0.01, ***P < 0.001. *IL-1β* interleukin-1 beta, *IL-6* interleukin-6, *LPS* lipopolysaccharide, *SOCS3* suppressor of cytokine signaling 3, *TNF-α* tumour necrosis factor alpha

After having established that the *CXCL10* promoter can mediate regulated expression in OA synovial micromasses, we set out to determine whether the transgene IL-10 can be a potential candidate for local treatment of OA. We first determined if IL-10 can lead to suppressor of cytokine signaling 3 (SOCS3) expression in the synovial micromasses. After 2 h stimulation with recombinant IL-10, SOCS3 was significantly upregulated (Fig. 3a). In addition, SOCS3 expression could also be induced by LPS and TNF-α as previously described [21]. Stimulation of the micromasses with LPS and TNF-α for 4 h resulted in significant upregulation of TNF-α and IL-1β mRNA (Fig. 3b,c). In LPS-stimulated conditions, IL-10 could reduce this induction. IL-6 was also upregulated after pro-inflammatory stimulation (Fig. 3c), but IL-10 could not reduce IL-6 expression. These results show that cells from the synovial micromass are responsive to IL-10 and downregulate the production of cytokines after stimulation, possibly via the induction of SOCS3.

**Fig. 4:**
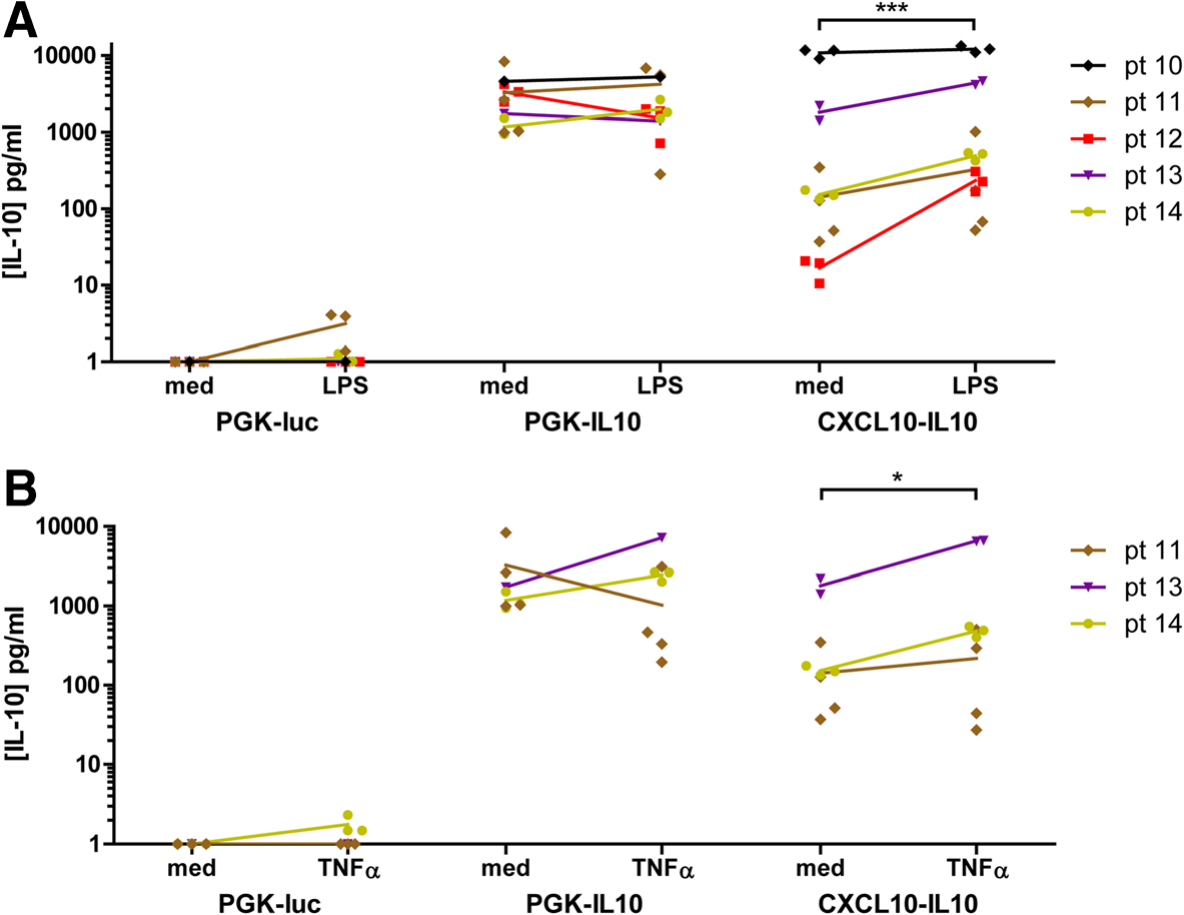
Production of IL-10 by transduced micromasses. Micromasses from synovial cell suspensions were transduced with lentiviral vectors coding for PGK-luciferase, PGK-IL10 or CXCL10p-IL10 after formation of a synovial lining. Subsequently, the micromasses were stimulated with medium containing 100 ng/ml LPS (**a**) or 10 ng/ml TNF-α (**b**). The IL-10 concentration in the supernatant after 24 h was measured using a multiplex ELISA assay. Concentrations below 1 pg/ml were included in the statistical analysis, but are shown as 1 pg/ml in the Figure. An insufficient number of micromasses could be generated from patients 10 and 12 to determine the response to TNF-α. The medium, LPS- and TNF-α-stimulated groups were compared by one-way ANOVA. *P < 0.05, **P < 0.01. *IL-10* interleukin-10, *LPS* lipopolysaccharide, *TNF-α* tumour necrosis factor alpha

We subsequently determined if the inducibility of the *CXCL10* promoter can be used to provide relevant IL-10 levels in synovial micromasses. After formation of the lining, the micromasses were transduced with IL-10 lentiviruses under control of the constitutive active *PGK* promoter or the inducible *CXCL10* promoter. The IL-10 levels in the supernatant of the micromasses were determined after 24 h stimulation with LPS and TNF-α. Micromasses transduced with PGK-luciferase control virus only produced low quantities of IL-10, indicating that the background production of endogenous IL-10 is low, even after stimulation with LPS or TNF-α (Fig. 4). Under control of the *PGK* promoter, high levels of IL-10 were secreted into the supernatant, which did not significantly differ after stimulation with LPS or TNF-α. In contrast, micromasses transduced with CXCL10p-IL10 showed an increase in IL-10 production after stimulation with either LPS or TNF-α.

**Fig. 5:**
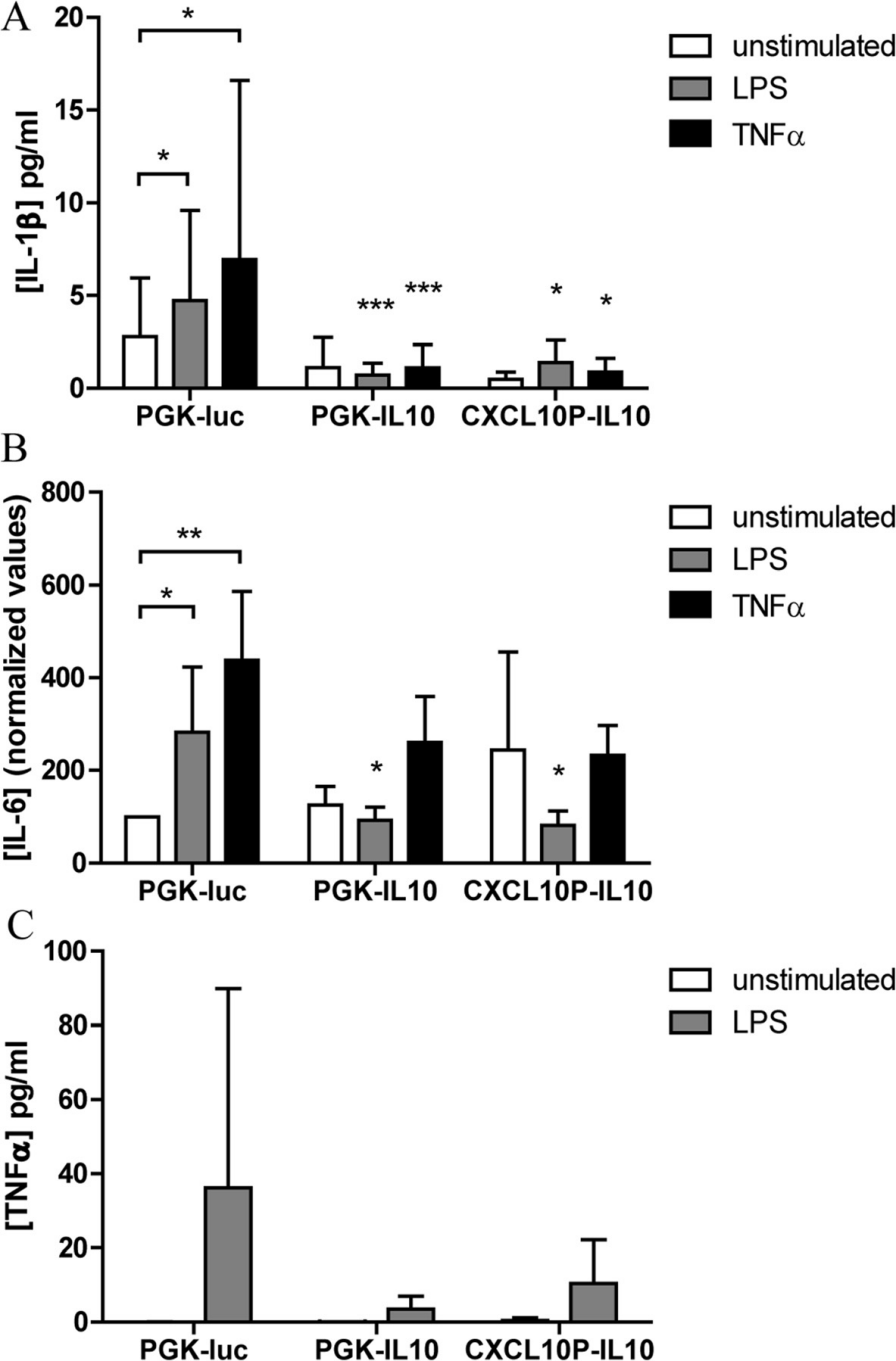
Cytokine production by stimulated micromasses treated with IL-10 viral vectors. Micromasses from synovial cell suspensions of five patients were transduced with lentiviral vectors coding for PGK-luciferase, PGK-IL10 or CXCL10p-IL10 after formation of a synovial lining. Subsequently, the micromasses were stimulated with medium containing 100 ng/ml LPS (all patients) or 10 ng/ml TNF-α (three patients). The concentrations of IL-1β (**a**), IL-6 (**b**) and TNF-α (**c**) in the supernatant after 24 h were measured using a multiplex ELISA assay. IL-1β and TNF-α could only be quantified in three patients. Because of high variations in IL-6 production between patients, the values were first normalized for every individual patient for PGK-luc unstimulated. The basal values (pg/ml) were 2.7 × 10^5^, 6.5 × 10^5^, 2.7 × 10^4^, 5.1 × 10^4^ and 3.3 × 10^5^ respectively. The medium, LPS- and TNF-α-stimulated groups were compared by *t* test. Significancies without a *capped line* were compared to PGK-luc. *P < 0.05, **P < 0.01, ***P < 0.001. *IL-1β* interleukin-1 beta, *IL-6* interleukin-6, *LPS* lipopolysaccharide, *TNF-α* tumour necrosis factor alpha

Next, we used the micromass culture supernatants to investigate if the IL-10 production from the transduced micromasses can influence the inflammatory response in the synovial lining. Under control conditions without stimulation, the micromasses already produced the pro-inflammatory cytokines IL-1β and IL-6 (Fig. 5a,b). The production increased after stimulation with LPS or TNF-α, which has also been observed in vivo [22]. The stimulatory effects of LPS and TNF-α on the release of IL-1β could be abolished by both constitutive (84.8 % after LPS and 83.9 % after TNF-α) and inducible (70.7 % after LPS and 87.4 % after TNF-α) expression of IL-10. Similar effects were observed for LPS-induced IL-6 secretion (Fig. 5b). After stimulation with LPS, the IL-6 production was decreased by 67.1 % by PGK-IL10 and 71.0 % by CXCL10p-IL10. TNF-α was below detection limits in multiple unstimulated micromasses (Fig. 5c). After stimulation with LPS, TNF-α was produced by the micromasses, but virus treatment showed no significant effects. These results show that treatment of a synovial lining with the inducible *CXCL10* promoter for the expression of IL-10 can reduce the production of pro-inflammatory cytokines and can inhibit stimulation of the lining.

## Discussion

In this study we set out to study the therapeutic potential of disease-inducible gene therapy for OA using the CXCL10p-IL10 lentivirus in a synovial lining model resembling early-stage OA that includes both synovial fibroblasts and synovial macrophages. After digestion of a synovial tissue sample the cells were mixed with Matrigel to obtain a synovial micromass, in which both FLS and MLS migrated to the micromass edge to form a lining. After transduction with a lentiviral CXCL10-promoter reporter vector, the *CXCL10* promoter could be significantly induced by pro-inflammatory stimulation. When the *CXCL10* promoter drove the expression of the gene coding for anti-inflammatory IL-10, sufficient IL-10 could be produced to reduce the release of IL-1β and IL-6 after stimulation with TNF-α or LPS.

In vitro studies with human synovial cells are often performed in monolayer culture, in which the cells might behave and interact differently from the complex structural organization in the joint [23]. To improve in vitro synovium studies, Kiener at al. developed a 3D synovial lining model [24]. Primary FLS are mixed with Matrigel to form a micromass structure, in which the FLS migrate towards the outside to form a lining layer, a phenomenon not observed with dermal fibroblasts. The micromass lining shows many similarities to the synovial lining. It expresses the synovial fibroblast marker proteoglycan 4 (PRG4) and produces extracellular matrix [25]. MLS were not included in these studies, but the FLS could support the survival of monocytes from peripheral blood.

Interestingly, macrophages in culture without the appropriate stimulation undergo apoptosis [26]. Kiener et al. showed that primary monocytes from the blood are not viable in micromasses when cultured alone, but could survive for over 3 weeks when cultured together with FLS [25]. We showed that the micromasses can also support the survival of synovial macrophages and that the macrophages resided in the lining. Because the micromasses do not show the fibrotic changes and macromolecular cartilage and bone detritus-rich synoviopathy observed in late-stage OA, the synovial micromass might more closely mimic early-stage OA compared to synovial explants from joint replacement remnants. This enables the possibility to test therapeutic strategies in an early disease stage where inhibition of inflammation might prevent disease progression.

During inflammation, synovial macrophages become activated and produce pro-inflammatory cytokines, matrix-degrading enzymes and promote synovial hyperplasia [27]. The synovial micromass model based on primary tissue digestions can be a suitable model to study these processes. The inclusion of MLS in the micromasses is particularly important for testing IL-10-based therapies, since the primary receptor subunit for IL-10, IL-10 receptor subunit alpha (IL-10R1), is most strongly expressed on cells of hematopoietic origin [28, 29]. After transduction of the micromasses with lentiviral PGK-GFP, transgene expression was predominantly observed in the membrane, which is similar to observations with intra-articular injections of lentivirus in vivo [30], providing further support that micromasses are an adquate model for the synovial membrane.

IL-10 is a powerful anti-inflammatory cytokine and IL-10-based gene therapy has proven to be effective in multiple animal models of OA and cartilage damage, alone and in combination with IL-1 receptor antagonist (IL-1Ra) or interleukin-4 (IL-4) [31, 32]. Low innate production of IL-10 by blood cells upon lipopolysaccharide (LPS) stimulation ex vivo is associated with an increased risk of OA [33]. In addition, it has been concluded from a systematic review that physical exercise has positive effects on pain and disability in knee OA [34]. These effects might be mediated by IL-10, which was found to be upregulated in the synovial fluid after exercise [35]. In addition to the inhibition of inflammation and matrix-degrading enzymes, IL-10 has chondroprotective and anabolic effects on cartilage by stimulating chondrocyte proliferation, stimulating the production of extracellular matrix components and reducing chondrocyte apoptosis [2, 36]. One of the mechanisms by which IL-10 can exert its anti-inflammatory effects, is by inducing the expression of SOCS3 [37]. SOCS3 can inhibit Janus kinase signal transducer and activator of transcription (JAK-STAT) signaling induced by cytokine receptor activation [38]. A second anti-inflammatory mechanism proposed for IL-10 is the inhibition of proteins that stabilize TNF-α messenger RNA (mRNA) [39]. As a result, the TNF-α mRNA becomes prone to degradation mediated by the 3′UTR AU-rich elements (AREs). We have found both upregulation of SOCS3 and reduced levels of TNF-α mRNA after treatment with recombinant IL-10, indicating that IL-10 might inhibit the inflammatory response in synovial micromasses at multiple levels.

The levels of TNF-α, IL-1β and IL-6 are increased in OA patients and high levels are associated with increased radiographic progression [40]. We have observed increased production of these cytokines by the micromasses under inflammatory conditions. TNF-α and IL-1β mRNA could be downregulated by IL-10 and the produced levels of IL-1β and IL-6 decreased after treatment with both PGK-IL10 and CXCL10p-IL10. Interestingly, the variation in basal IL-10 production between patients after transduction with CXCL10p-IL10 was bigger compared to the IL-10 production after stimulation with LPS or after transduction with PGK-IL10 (Fig. 4). The variation in basal IL-10 production from the CXCL10p-IL10-transduced micromasses might result from a variation in tissue composition or differences in inflammatory imprinting of synovial cells between patients rather than transduction efficiency, because less variation in IL-10 production after transduction with PGK-IL10 was observed.

## Conclusions

We showed that 3D micromasses that are made from primary synovial cells of OA patients form a synovial lining consisting of fibroblast-like and macrophage-like synoviocytes and the micromasses might be a useful tool to study synovium in vitro. The micromass lining can be transduced by lentiviruses and provide disease-inducible expression of sufficient amounts of IL-10 to reduce the production of pro-inflammatory cytokines by the micromasses. Gene therapy with the CXCL10p-IL-10 vector might be a promising strategy for local treatment of early OA.

## Abbreviations

2D, two-dimensional; 3D, three-dimensional; ANOVA, analysis of variance; ARE, AU-rich element; CD68, cluster of differentiation 68; CXCL10, C-X-C motif chemokine 10; DAMP, damage-associated molecular pattern; DAPI, 4′,6-diamidino-2-phenylindole; ELISA, enzyme-linked immunosorbent assay; FCS, fetal calf serum; FLS, fibroblast-like synoviocytes; GFP, green fluorescent protein; HEMA, 2-hydroxyethyl methacrylate; HRP, horseradish peroxidase; H&E, hematoxylin and eosin; IL-1RA, interleukin-1 receptor antagonist; IL-1β, interleukin-1 beta; IL-4, interleukin-4; IL-6, interleukin-6; IL-10, interleukin-10; IL-10R1, IL-10 receptor subunit alpha; JAK-STAT, Janus kinase signal transducer and activator of transcription; LPS, lipopolysaccharide; MLS, macrophage-like synoviocyte; mRNA, messenger RNA; MCS, multiple cloning site; OA, osteoarthritis; PBS, phosphate-buffered saline; PGK, phosphoglycerate kinase; PRG4, proteoglycan 4, lubricin; qPCR, quantitative polymerase chain reaction; RA, rheumatoid arthritis; RLU, relative light units; RPL22, 60S ribosomal protein L22; RPMI, Roswell Park Memorial Institute; SOCS3, suppressor of cytokine signaling 3; TNF-α, tumour necrosis factor alpha

## Additional files

Additional file 1: Figure S1. HE stainings of 7 μm cryosections obtained from patient biopsies used in this study. (TIF 15170 kb) Additional file 2: Figure S2. Scatter plots of flow cytometry analysis of micromasses transduced with PGK-GFP. Seven days after formation, the micromasses were transduced with lentiviral PGK-GFP. Forty-eight hours after transduction, the micromasses were melted and stained for FLS (11-Fibrau) and MLS (CD68) markers. Cells were first gated for the live gate. (**A**) GFP signal and forward scatter (**B**) GFP signal and 11-Fibrau signal (**C**) GFP signal and CD68 signal. The scatter plots are representative for multiple experiments. (TIF 2114 kb)
