# Supplementary figures and images for: Suppression of the inflammatory response by disease-inducible interleukin-10 gene therapy in a three-dimensional micromass model of the human synovial membrane
Source: Arthritis Res Ther. 2016 Aug 12;18:186. doi: 10.1186/s13075-016-1083-1 (PMC4983024; doi:10.1186/s13075-016-1083-1)

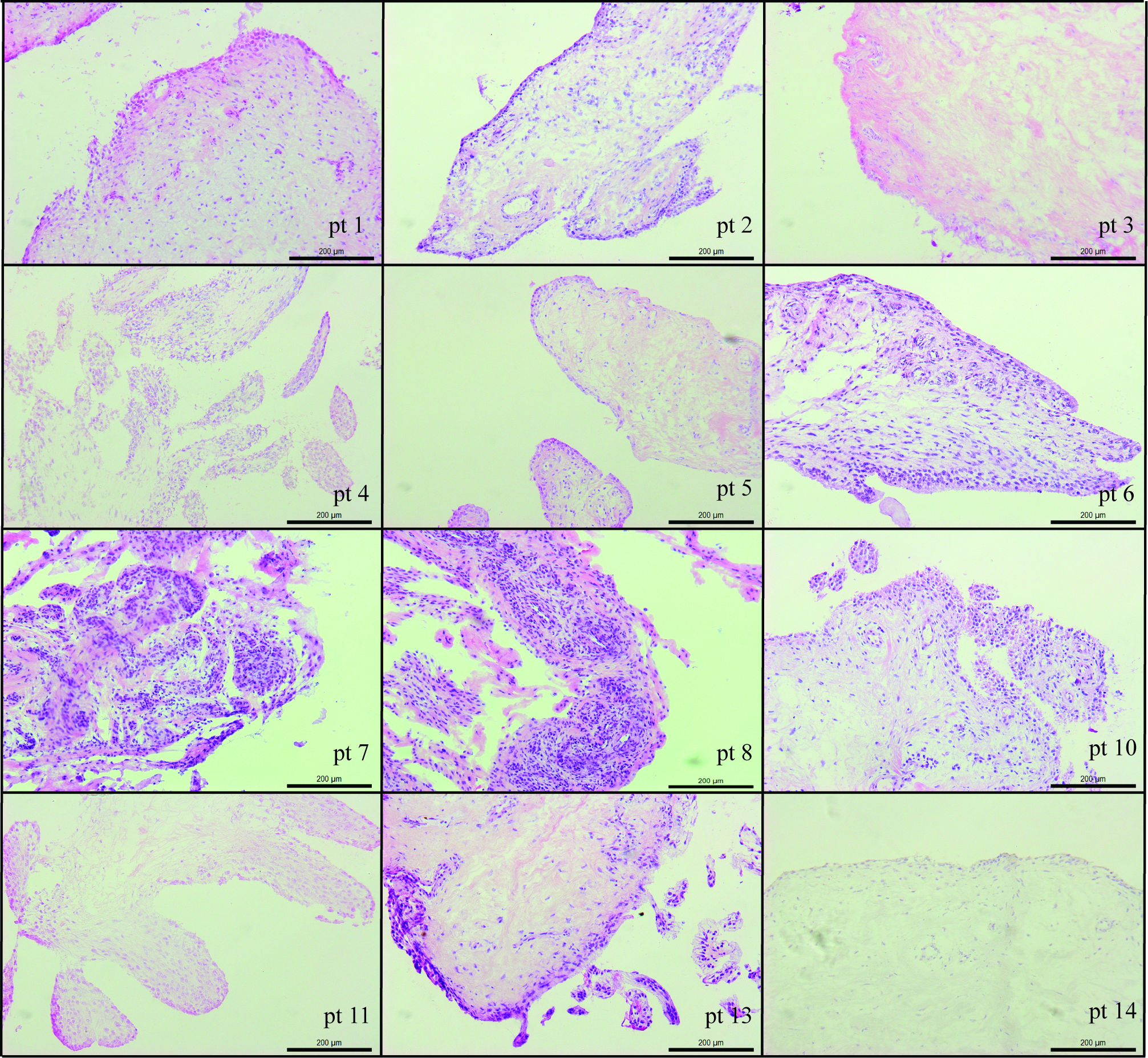

Supplement: Additional file 1: Figure S1. — HE stainings of 7 μm cryosections obtained from patient biopsies used in this study. (TIF 15170 kb) [file 13075_2016_1083_MOESM1_ESM.tif]

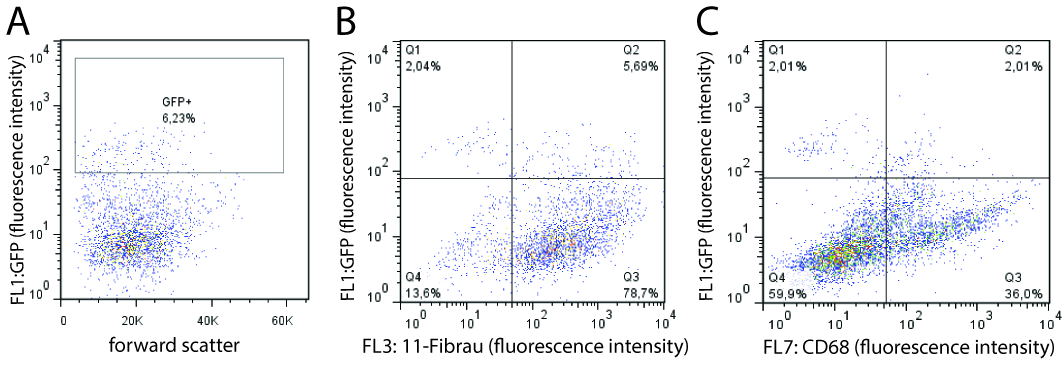

Supplement: Additional file 2: Figure S2. — Scatter plots of flow cytometry analysis of micromasses transduced with PGK-GFP. Seven days after formation, the micromasses were transduced with lentiviral PGK-GFP. Forty-eight hours after transduction, the micromasses were melted and stained for FLS (11-Fibrau) and MLS (CD68) markers. Cells were first gated for the live gate. (A) GFP signal and forward scatter (B) GFP signal and 11-Fibrau signal (C) GFP signal and CD68 signal. The scatter plots are representative for multiple experiments. (TIF 2114 kb) [file 13075_2016_1083_MOESM2_ESM.tif]
